# Psychometric properties of the thyroid-specific quality of life questionnaire ThyPRO in Singaporean patients with Graves’ disease

**DOI:** 10.1186/s41687-021-00309-x

**Published:** 2021-07-08

**Authors:** Huiling Liew, Torquil Watt, Luo Nan, Alvin W. K. Tan, Yiong Huak Chan, Daniel Ek Kwang Chew, Rinkoo Dalan

**Affiliations:** 1 Department of Diabetes and Endocrinology, Tan Tock Seng Hospital, 11 Jalan Tan Tock Seng, Singapore, 308433 Singapore; 2 Department of Medical Endocrinology, Copenhagen University Hospital Rigshospitalet, Copenhagen, Denmark; 3 Saw Swee Hock School of Public Health, National University of Singapore, Singapore, Singapore; 4 Biostatistics Unit, Yong Loo Lin School of Medicine, National University of Singapore, Singapore, Singapore; 5 Lee Kong Chian School of Medicine, Nanyang Technological University, Singapore, Singapore; 6 Yong Loo Lin School of Medicine, National University of Singapore, Singapore, Singapore

**Keywords:** Hyperthyroidism, Quality of life, ThyPRO, Graves’, Asian

## Abstract

**Background:**

Graves’ disease is the most common cause of hyperthyroidism. It results in accelerated tissue metabolism with multi-organ involvement ranging from cardiovascular to neuropsychological function. This results in a negative impact on the quality of life (QOL) of the individual patient. We aim to evaluate the psychometric properties of ThyPRO, a Thyroid-related Patient Reported Outcome questionnaire, and validate its use in our multi-ethnic Asian patients with Graves’ hyperthyroidism.

**Methods:**

Forty-seven consecutive Graves’ hyperthyroidism patients answered the ThyPRO questionnaire at baseline and at 4 months after treatment initiation. Data were recorded for thyroid related symptoms and signs, thyroid function tests and thyroid volume. We analyzed the internal consistency using Cronbach’s alpha, construct validity by evaluating relationship between clinical variables and ThyPRO scales, ceiling and floor effects, and responsiveness of ThyPRO to treatment based on Cohen’s effect size.

**Results:**

Correlations between individual scale scores and free thyroxine concentrations were moderate and statistically significant: 0.21–0.64 (*p* <  0.05). There was high internal consistency between the items in this instrument, Cronbach’s alpha > 0.7 for all scales. ThyPRO was responsive to the changes in QOL after treatment (Effect Size: 0.20–0.77) in 9 of the 14 scales including the hyperthyroid symptoms and psychosocial scales (Tiredness, Cognitive complaints, Anxiety, Emotional susceptibility, Impact on Social, Daily and Sex life).

**Conclusion:**

This study provides evidence that ThyPRO has satisfactory measurement properties in hyperthyroid Graves’ disease patients in Singapore population with the potential to complement clinical care.

**Supplementary Information:**

The online version contains supplementary material available at 10.1186/s41687-021-00309-x.

## Plain English summary

### What is the key problem/ issues/ question this manuscript addresses?

Graves’ disease is the most common cause of hyperthyroidism. It negatively affects the patients’ ability to perform activities ranging from daily living to work. Yet in a busy clinic, doctors seldom have time to collect information on these aspects which are meaningful to the patients.

### Why is this study needed?

It is important to evaluate the functional and social aspects of hyperthyroid Graves’ disease patients in Singapore population. These experiences varied in different countries and culture.

### Main point of my study

In this study, we evaluate the accuracy of a thyroid-specific quality of life questionnaire ThyPRO in measuring the health aspect of Graves’ hyperthyroidism in Singapore patients and correlate with their thyroid blood tests, thyroid volume and complaints. We repeated the questionnaire 4 months after anti-thyroid medications to gauge the responsiveness of the survey.

### Brief overview of my results and what they mean

This study demonstrates that the survey scores correspond with improvement in the thyroid blood tests. The questionnaire also shows how Graves’ disease patients in Singapore population related to their functions including tiredness, emotions and activities of living after starting antithyroid medication. This result encourages future research on our population’s experiences with different treatment options (anti-thyroid medication, radioactive therapy, surgery) hence potential to improve clinical care.

## Introduction

Hyperthyroidism is a prevalent clinical condition with a reported global prevalence of about 1% [1]. Graves’ disease, the most common cause of hyperthyroidism [2, 3], is an immunologically-mediated condition where the thyroid gland is stimulated by thyroid receptor antibodies with a resultant increased synthesis of thyroid hormones [4, 5]. In the untreated state, the rise in circulating thyroxine levels accelerates tissue metabolism. It results in multi organ involvement resulting in significant impact on the eye, neuropsychology, cardiovascular, gastrointestinal, and neurological systems [6]. The accompanying negative influence on the individual’s well-being and social functioning is well known, including increased somatic and psychiatric morbidity such as anxiety, depression, work disability and mortality [1, 7–16].

Graves’ disease is usually managed in the ambulatory setting with individualized treatment with either anti-thyroid medications, radioactive iodine, or surgery. Patients with Graves’ disease do not feel completely well despite normalization of thyroid function [17–20]. Compared to other causes of hyperthyroidism, patients with Graves’ hyperthyroidism experience higher levels of anxiety and depression [18, 19]. Studies have shown that the hyperthyroid symptoms are correlated to the size of thyroid goitre, not the thyroid hormone concentration [21]. Despite being cognizant of the negative influence of the hyperthyroid status on various aspects of living, clinicians seldom have the time to probe the impact of hyperthyroidism on the individual’s quality of life (QOL). These reasons underscores the need to optimise the health-related QOL of patients with Graves’ diseases besides focusing on their clinical signs and biochemical tests [22, 23].

Health related QOL surveys are broadly classified into generic questionnaire, which looks at the general aspects of QOL in the population, and disease-specific questionnaire, which relates particularly to a medical condition [22, 24]. To assess the QOL of patients with Graves’ disease, it is important to use a disease-specific questionnaire that measures the intended patient reported outcomes (PRO) of their experience of the disease and its impact on their well-being. This property of evaluating the accuracy to which the questionnaire measures the concept of interest in a target population is known as validity: content validity examines the respective items in a scale for its relevance and coverage, whereas construct validity inspects the relationship of the items, scales to a priori hypothesis [25, 26]. Importantly, the instrument for evaluating PRO has to be reproducible, which means yielding the same result on independent repeat assessments, and be responsive in terms of its ability to detect meaningful changes in the measured QOL with treatment [22, 24, 27].

Of the instruments targeting population with hyperthyroidism, Hyperthyroidism Complaint Questionnaire and Thyroid specific patient reported outcome (ThyPRO) questionnaire were identified. The measurement properties of ThyPRO was the most published with proven hypothesis testing, internal consistency, reliability, content validity, structural validity and responsiveness [24, 25, 27–29]. In Danish speaking patients, ThyPRO has been rated good to excellent in its measurement properties. Also, it has cross -cultural validity in many languages [30–35].. To improve the ease of administration, the developer has also shortened the original questionnaire from 84 to 39 questions in 13 scales, ThyPRO-39 [36].

ThyPRO has been used in studies looking at the QOL of subjects with various benign thyroid conditions [37–39], in randomized control trials looking at the QOL of Graves’ patients on selenium supplementation [40], post thyroidectomy [41], post radioactive-ablation [42], and in patients with non-functioning thyroid nodules after percutaneous laser ablation [43]. There is an ongoing study looking at the improvement in QOL of post-radioactive ablated Graves’ patients who were rendered euthyroid with levothyroxine dose towards a target thyroid function range based on mathematical set point theory [44].

According to World Health Organisation global database on iodine deficiency, South East Asia region has iodine status ranging from predominantly optimal to mild insufficiency [45]. In iodine-replete countries like Singapore, the thyroid disorders are mainly autoimmune conditions such as Graves’ disease and Hashimoto’s thyroiditis [2]. There is paucity of literature on patient reported outcomes on benign thyroid conditions in Singapore and Southeast Asia. Singapore is a unique country with a multi-ethnic 5.69 million residents (74.3% Chinese, 13.5% Malays, 9.0% Indians and 3.2% other ethnicity) [46] . English is the main language and majority of the population is bilingual [47]. Besides being culturally diverse, Singapore, being near the Equator and part of South East Asia, has a tropical rainforest climate: constant warm and humid with daily temperature ranging between 25 °C to 33 °C henceforth native subjects with Graves’ hyperthyroidism may perceive heat intolerance and the impact of the disease differently from elsewhere.

The purpose of this study was to evaluate the psychometric properties of ThyPRO among Singaporean patients with Graves’ disease after treatment with anti-thyroid medication.

## Materials and methods

### Patients and study design

We enrolled 47 patients (35 Females; 12 Males) with Graves’ hyperthyroidism consecutively from a tertiary endocrine centre in Singapore between March 2014 and February 2015. This study was conducted in accordance with the declarations of Helsinki and approval was obtained from the institutional review board (IRB) [National Healthcare Group Domain Specific Review Board (DSRB) Reference No. 2012/ 01098]. Written informed consent was obtained from all subjects.

The inclusion criteria were: [1] age: 21 to 80 years old, [2] able to read and understand English and [3] biochemical hyperthyroidism (defined as free thyroxine (FT4) above upper limit of normal with our laboratory reference range for normal FT4 being 8 to 21 pmol/L, and thyrotropin (TSH) suppressed below low limit of normal with our laboratory reference range for normal TSH being 0.34 to 5.60 mIU/L, at time of recruitment. The exclusion criteria were: [1] cognitive impairment, [2] biochemically euthyroid status [3] pregnancy.

TSH receptor antibodies (TRAb) were performed in all subjects for establishment of the diagnosis of Graves’ disease. In one case where the TRAb was negative, Thyroid Stimulating Immunoglobulin TSI assay was sent. Ultrasound measurement of the thyroid was done, and volume measurement of each thyroid lobe was obtained with the General Electric Logiq P5 ultrasound machine using the Brunn formula: width (cm) x length (cm) x depth (cm) × 0.479 [48]. The thyroid volume is the summation of the two thyroid lobes.

Clinical data including patients’ demographics, clinical symptoms, physical examination, vital signs, diagnosis, and treatment were collected via the standardised electronic clerking template in the clinic as per routine clinical care. Clinical symptoms were categorized as follows: general, cardiorespiratory, neurological, gastrointestinal, eye and constitutional (Supplementary I, Table 1). The subject was considered to have the group of symptoms if he/she has at least one symptom in that category. At 4 months after initiation or titration of antithyroid medications (either carbimazole or propylthiouracil), the TFT, ultrasound and the ThyPRO questionnaire were repeated. Five patients were lost to follow-up and answered only one baseline questionnaire. Thiamazole dose tabulation was converted to carbimazole dose using an equivalence of 0.6 to 1.0 [6].

### Instruments

#### ThyPRO

ThyPRO is a questionnaire which evaluates the impact of benign thyroid disease on QOL, developed by Watt et al [22, 25, 29]. It is a self-administered questionnaire with 84 items within 13 scales and one overall QOL-impact scale. Higher scores implicate more negative symptoms and worse thyroid-related QOL. The questions relate to physical symptoms of Goitre, Hyperthyroid, Hypothyroid, and Eye Symptoms; psychological symptoms of Anxiety, Depressivity; Functional and well-being symptoms of Tiredness, Cognitive Complaints, Emotional Susceptibility; its impact on participation in activities of Social Life, Sex Life and Daily Life; Cosmetic Complaints; and the overall impact on the QOL. The patients’ responses are based on how they have been feeling on a 4-week retrieval period [27]. In accordance with ThyPRO standard scorings, each item is scored on a 5-point Likert scale (0=” not at all”, 4=” very much” or “completely”). Each scale is derived by summation of relevant item (after reversal of positively worded items) and linear transformation to 0–100 [25]. The English version of the ThyPRO is administered at baseline and at 4 months in this study.

#### Local clinicians’ review

Prior to commencement of the study, 9 local experienced peer and/or senior endocrinologists reviewed the questionnaire and examined the feasibility and applicability of the questions in our local context based on their clinical experience (yes/ no/ not sure). The survey showed that three quarters of them agreed that more than 80% of the items in the questionnaire were applicable to our local patients.

### Data analysis

Data was analysed using STATA version 14.0 (College Station, Texas 77,845, USA) with statistical significance set at *p* <  0.05. Descriptive data for numerical variables were presented as mean (Standard Deviation SD) and n (%) for categorical variables and median (Interquartile Range IQR) for ordinal variables. Paired t-test was performed to compare thyroid function variables, thyroid volumes and carbimazole doses at baseline and at 4 months, when normality assumptions were satisfied, otherwise Wilcoxon signed rank test was used.

The construct validity of this ThyPRO tool was assessed using the relevant ThyPRO scales with the categories of presenting symptoms, thyroid function and thyroid volume [22, 28].

We hypothesised that ThyPRO hyperthyroid and goitre scale correlated with the thyroid function tests and thyroid volume respectively and that the more abnormal the thyroid function, the worse is the QOL assessing the social and function wellbeing scales (Tiredness, Cognitive Complaints, Anxiety, Depressivity, Impaired Social life, Impaired Daily life, Impaired Sex Life, Cosmetic Complaints, and Overall QOL Impact). Item-test correlation was performed using Pearson correlation for parametric variables; otherwise, Spearman correlation was used. A correlative coefficient value of 0.5 or higher, 0.35–0.5, and 0.2–0.35 would be considered strong, moderate, and weak correlation respectively [49].

Cronbach’s alpha was used to assess internal consistency reliability where a value of above 0.7 indicates satisfactory degree of internal consistency [25, 28, 50]. Cohen’s effect size, defined as the mean change of each item scale score from baseline to 4 months over the standard deviation, was used to evaluate the responsiveness of the questionnaire to treatment. Cohen’s effect size of greater than 0.8 was considered large, between 0.5 to 0.8 moderate otherwise small [51]. We sought to look at the responsiveness in two aspects: [1] whether the change in QOL scales is clinically significant based on Cohen’s effect size and [2] whether the change in QOL correlates with the change in thyroid function tests and thyroid volume.

In terms of evaluating the minimally important change (MIC), a one-way ANOVA was conducted to determine if the mean change in negative QOL was different for groups with different categories of negative impact on overall QOL. They were classified into five groups (“Not at all”, “A little”, “Some”, “Quite a bit”, and “Very much”) based on their overall negative QOL score at 4 months where the higher the score, the more negative impact the thyroid condition has on their QOL.

## Results

### Patient characteristics

At baseline, 47 subjects with Graves’ hyperthyroidism completed the self-administered English questionnaire with ease. The majority were females (35, 74%), Chinese (43, 92%) and non-smokers (29, 70%). All subjects were bilingual in English and either Chinese or Malay. Approximately, half (24/47, 53%) of them had family history of thyroid disorders. The mean age of diagnosis was 38.5 ± 14.1 years old. Table 1 depicts the baseline demographic, clinical and thyroid ultrasound characteristics of the 47 subjects. The mean FT4 was 45.9 ± 22.0 pmol/L (RI: 8.0–21.0) and median TSH was 0.02 (0.01–0.03) mIU/L (RI: 0.34–5.60). The mean TRAb concentration was 16.0 ± 13.3 IU/L (Table 2). At 4 months of follow up, the FT4 normalised in 90% (38/42) and TSH remained suppressed in 64% (27/42) of the individuals. The improvement in thyroid function and volume was statistically significant (*P* < 0.05) (Table 2).

**Table 1:** Demographic and clinical features of patients

Characteristics	Subjects (*n* = 47)	
Female, *n* (%)	35	(74.5)
Chinese, *n* (%)	43	(91.5)
Malay, *n* (%)	4	(8.5)
Smoking, *n* (%)		
Non-smoker	29	(69.1)
Ex-smoker	9	(20.5)
Current smoker	4	(9.1)
Presence of family history of thyroid disease, *n* (%)	24	(53.3)
Mean age at diagnosis, *years* (SD)	38.5	(14.1)
Mean duration of condition, *years* (SD)	5.5	(10.2)
Immunological diagnosis		
Thyrotropin Stimulating Hormone Receptor Antibody Positive	46	(97.9)
Thyroid Stimulating Immunoglobulin Positive	1	(2.1)
**Symptoms of hyperthyroidism**		
General, *n* (%)	31	(67.4)
Cardiorespiratory, *n* (%)	34	(72.3)
Gastrointestinal, *n* (%)	33	(70.2)
Neurological, *n* (%)	28	(59.6)
Eye, *n* (%)	12	(25.5)
Constitutional, *n* (%)	14	(29.8)
**Signs of hyperthyroidism**		
Heart rate, *bpm* (SD)	91	(21.6)
Goitre, *n* (%)	28	(62.2)
Clinical hyperthyroidism, *n* (%)	31	(70.5)
**Biochemical and Ultrasound**		
Mean free thyroxine, *pmol/L* (SD)	45.9	(22.0)
Median TSH, *mIU/L* (IQR)- Range 0.00249–0.22	0.02	(0.01–0.03)
Mean TRAb, *IU/L* (SD)	16.0	(13.3)
**Thionamide treatment**		
Propylthiouracil, *mg/day* (SD)	90	(42)
Carbimazole, *mg/day* (SD)	16	(8)

**Table 2:** Biochemical and ultrasound thyroid volume at first visit and at 4 months later

*n* = 42, Longitudinal follow-up		Baseline	4 months later	*P* value
		Mean (SD)/ Median (IQR)		
**Thyroid function and immunological markers**				
**Thyroxine Free**	pmol/L	45.8 ± 22.2	15.6 ± 11.3	< 0.0001
**TSH**	mIU/L	0.03 (0.02–0.04)	1.42 (0.32–2.54)	0.0147
**Thionamides**				
**Carbimazole, mg/day**	Dose	15.9 ± 8.5	11.2 ± 7.4	0.0006
**Propylthiouracil, mg/day**	Dose	90.0 ± 41.8	90.0 ± 54.8	1.000
**Sonographic Volume**				
**Thyroid volume**	Cc	20.1 ± 12.3	15.5 ± 9.7	0.023

*SD* Standard deviation, *IQR* Interquartile range, *pmol/L* picomoles per litre, *mIU/L* milli international units per litre, *mg/day* milligram per day, *Cc* Cubic centimetre

#### ThyPRO evaluation

##### Distribution approach

The distributional characteristics of ThyPRO are shown in Table 3. For the ThyPRO completed by 47 subjects, only 6 items had a missing response which were imputed as the mean score of the scale if half or more of the items in the scale were filled. There were no missing data for the follow-up questionnaires. There was no ceiling effect (defined as more than 15% of subjects attaining the maximum score of 100) observed in all the scales.

**Table 3:** Distribution characteristics of ThyPRO score at baseline

Scales, ***n*** = 47 observations	No. of items	Mean (SD)	Median (Interquartile range)	Min - Max range	Floor/ Ceiling (%)	Cronbach’s alpha
**ThyPRO Original, Baseline**						
**Goitre Symptoms**	11	12 (13)	7 (2–20)	0–45	20/0	0.81
**Hyperthyroid Symptoms**	8	37 (25)	34 (19–67)	0–78	4/0	0.87
**Hypothyroid Symptoms**	4	24 (24)	19 (6–27)	0–100	17/ 2	0.75
**Eye Symptoms**	8	15 (18)	7 (0–28)	0–75	33/0	0.87
**Tiredness**	7	50 (24)	52 (32–64)	0–100	2/4	0.85
**Cognitive Complaints**	6	23 (28)	13 (0–42)	0–100	31/4	0.95
**Anxiety**	6	30 (26)	25 (8–46)	0–100	17/2	0.91
**Depressivity**	7	29 (24)	29 (11–39)	0–100	10/2	0.91
**Emotional Susceptibility**	9	37 (21)	32 (22–53)	3–100	0/2	0.89
**Impact on Social life**	4	18 (21)	13 (0–25)	0–100	25/2	0.79
**Impact on Daily life**	6	20 (26)	8 (0–42)	0–100	40/ 2	0.93
**Impact on Sex life**	2	18 (30)	0 (0–25)	0–100	60/ 6	0.98
**Cosmetic Complaints**	6	20 (25)	8 (0–30)	0–96	25/ 0	0.85
**Overall QOL Impact**	1	33 (34)	25 (0–50)	0–100	31/13	–

Floor effects were noticeable in the following scales: Goitre symptoms, Hypothyroid symptoms, Eye symptoms, Cognitive complaints, Anxiety, Impact on Social life, Impact on Daily life, Impact on Sex life, Cosmetic complaints and overall quality of life impact (refer Table 3).

**Fig. 1:**
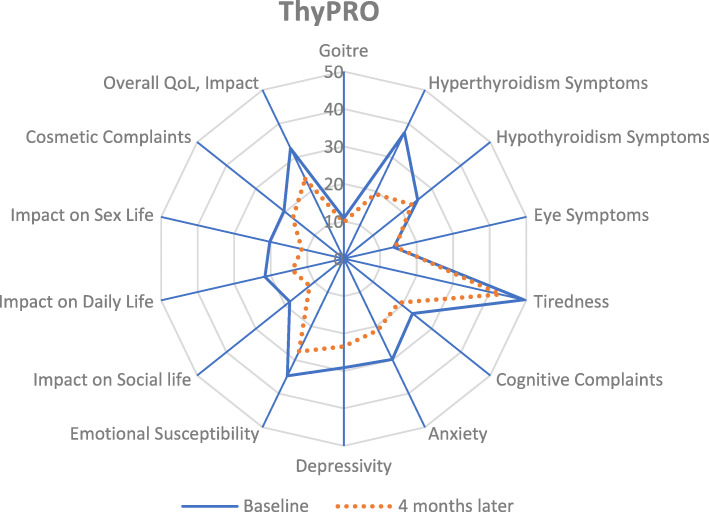
Radar plot of ThyPRO scale scores at baseline and 4 months later. The radar plot illustrated the improvement in the scales (range 0 to 100) of ThyPRO from baseline to 4 months after treatment of hyperthyroidism. The mean score of the following symptoms and psychosocial scales decreased after treatment signifying an improvement in the quality of life score: Hyperthyroidism symptoms, Tiredness, Anxiety, Emotional Susceptibility, Impact on Social Life, Impact on Daily Life and Impact on Sex Life. Legend: ThyPRO- Thyroid related Patient Reported Outcome

Supplementary II, Figure 1 illustrates histograms depicting the proportion of respondents across the range of score for each scale. Supplementary II, Figure 2 gives the breakdown of the items in the scale with floor effects.

#### Construct validity

All ThyPRO scales correlated with FT4 concentrations; of significance were the Hyperthyroid Symptoms scale with a correlation of 0.64 and the Anxiety scale 0.45 (*p* < 0.05). The Hyperthyroid Symptoms scale correlated with all the clinical symptoms except for constitutional symptoms. None of the scales correlated with thyroid volume. (Supplementary I, Table 2).

#### Reliability

Cronbach’s alpha exceeded 0.7 for all the scales in ThyPRO in the evaluation for internal consistency reliability (Table 3).

The item-test correlation was generally higher than 0.4 for all items except for items in the Goitre scale asking for “had pain in your neck that could be felt in your ears” (0.13), “had pain in front of your throat” (0.29), and in the Emotional susceptibility scale asking about whether one “felt in control of your life” (0.08), and “felt in balance” (− 0.02). (Supplementary I, Table 3).

#### Responsiveness

Out of the 47 subjects, 42 subjects completed the follow-up with a response rate of 90%. The Cohen’s effect size ranged from − 0.1 to 0.8 in ThyPRO (Table 4). The responsiveness was the highest for the hyperthyroidism symptom scale for both questionnaires. The improvement in the various scales in both ThyPRO was graphically illustrated in the radar plots in Fig. 1.

**Table 4:** Responsiveness in ThyPRO score after 4 months of treatment

Scale	ThyPRO				
	Baseline		At 4 months		Cohen’s Effect size
	Mean	SD	Mean	SD	
**Goitre Symptoms**	11.8	12.0	9.72	9.97	0.2^a^
**Hyperthyroid Symptoms**	36.8	25.5	19.2	20.2	0.8 ^b^
**Hypothyroid Symptoms**	26.7	24.4	23.2	21.6	0.2^a^
**Eye Symptoms**	13.4	16.4	14.2	13.8	−0.1
**Tiredness**	49.4	25.2	42.8	22.2	0.3^a^
**Cognitive Complaints**	24.9	29.7	18.8	23.0	0.2^a^
**Anxiety**	29.9	24.8	21.2	19.6	0.4^a^
**Depressivity**	27.6	24.6	23.5	15.7	0.2^a^
**Emotional Susceptibility**	34.5	23.3	27.5	17.5	0.3^a^
**Impact on Social Life**	18.8	22.4	11.6	17.9	0.4^a^
**Impact on Daily Life**	21.1	25.7	14.1	17.6	0.3^a^
**Impact on Sex Life**	18.9	30.7	11.4	21.5	0.3^a^
**Cosmetic Complaints**	19.9	25.0	17.4	20.7	0.1
**Overall QOL, Impact**	31.3	32.9	23.6	31.6	0.3^a^

Cohen’s Effect Size: 0.2 to 0.5 signifies small effect size^a^; 0.5 to 0.8 signifies moderate effect size^b^; above 0.8 signifies large effect size® Annotation based on rounded up to 1 decimal place

Thyroid experts anticipated the scales in shades to change with treatment

The hypothesized social and functioning well-being scales (Tiredness, Anxiety, Emotional Susceptibility, Impaired Social, Daily and Sex life scales in ThyPRO had moderate to large effect sizes as anticipated by the thyroid experts in a prior study [17].

##### Anchor based approach

At baseline, 14 (33%) subjects answered “Not at all” to negative impact on their quality of life. This proportion increased to 20 (47%) at 4 months. Those who were affected very much by their disease states decreased from 7 (16%) to 3 (7%). For those who chose “Very much” for baseline negative QOL, the mean change in score was an improvement of 46 points (Supplementary II, Figure 3a).

There was a close to statistically significant difference between groups as determined by one-way ANOVA (F (4,38) = 2.51, *p* = 0.0576). A pairwise comparison of mean test revealed that the negative QOL was statistically significantly higher in the category “Very much” compared to “Some” (83 ± 36, 95% CI 9.5–157.2, *p* = 0.028); “Very much” compared to “A little” (41 ± 20, 95% CI 0.9–81, *p* = 0.045); and “Very much” compared to “Not at all” (55 ± 20, 95% CI 15–94, *p* = 0.008). There were no statistically significant differences between the other groups (Table 5).

**Table 5:** Number and percentages of responses on the Negative QOL item at 4 months (Global Transition item) for different response categories, with effect sizes, mean score changes by response category, and ANOVA tests for linear trend for the ThyPRO

Measure	Not at all	A little	Some	Quite a bit	Very much
**Negative QOL 4 months later, Count (%) of responses**	20 (47)	16 (37)	1 (2)	3 (7)	3 (7)
**Mean change (SD)**	−21 (27)	−8 (33)	−50 (0)	0 (25)	33 (58)
**Effect size** ^**#**^	−13	reference	−42	8	41
***P*-value**	0.212	N.A.	0.203	0.696	0.045

Legend: %- Percentage; *SD* Standard deviation; Effect size takes “A little” as the reference

*N.A.* Not applicable

Using “A little” as the reference that distinguishes “Not at all” from the rest of the categories, the minimum, the MIC (Minimum Important Change or average score associated with “A little”) on the overall negative QOL scale at 4 months was − 8 points.

## Discussion

To our knowledge, this is the first study that comprehensively evaluated the psychometric properties of ThyPRO in our local population with Graves’ hyperthyroidism. Like previous studies, our study showed that ThyPRO fared well in six out of nine internationally agreed measurement properties namely internal consistency, reliability, content validity, structural validity, hypothesis testing and responsiveness.

Similar to Wong et al. who evaluated the measurement properties of ThyPRO-39 (Traditional Chinese version), there were significant (defined as > 15%) floor effects observed in 9 of the 13 domains namely Goitre, Hypothyroid symptoms, Eye symptoms, Cognitive complaints, Anxiety, Impact on Social life, Impact on Sex Life, Cosmetic Complaints, and Overall Impact [33]. There are 3 possible reasons for floor effects. Firstly, the majority of our subjects do not have all the clinical symptoms and only 25% of them had eye symptoms on clinical examination. This is consistent with a local paper where 70% of patients attending a thyroid eye clinic have mild symptoms [52]. These scales cover a comprehensive range of relevant symptoms for which some severe symptoms such as sensation of suffocation, impaired vision, double vision or eye pain are not commonly encountered for our patient population (Supplementary II, Figures 2a and 2c). Only 62% had a palpable goitre and on sonography, they were not very large. The range of Goitre symptoms score spanned between 0 to 45 with 20% of patients having the lowest possible score. It was possible that our cohort of patients had a higher QOL in this aspect than the scale where they did not experience globus sensation, pain, the need to clear throat, difficulty in swallowing, hoarseness or suffocation since they score mainly between the category of “not at all” to “a little”.

Secondly, the mean duration of the condition in our cohort was 5.5 years and some of the patients had relapses of their hyperthyroid conditions whilst on antithyroid treatment. The subjects may have become accustomed to their conditions hence they did not have significant impairment of their QOL in these aspects based on the questionnaire which used last 4 weeks recall. Thirdly, the relevance of items in a scale contribute to the floor effects in certain scales. In the Cosmetic scale, one of the items enquiring on whether the thyroid disease made the respondent feel too fat may not be relevant in our cohort of patients with hyperthyroidism (Supplementary II, Figure 2i). In this condition, subjects tend to lose weight.

Given the lower prevalence of reported anxiety symptoms in Singapore compared to Western countries [53], it was likely that our patient population minimizes their anxiety symptoms in our culture hence accounting for the floor effect of 17% in the Anxiety symptom scale. In terms of negative impact on social and daily life, the scale, it could be possible that Singaporeans have a higher QOL in these domains. This was substantiated by previous studies demonstrating a high quality of life in Singapore and the 2019 Mercer Global Quality of living report [54–56] In Watt et al., paper looking at thyroid -attributable impaired sex QOL, 57% of women with Grave’s hyperthyroidism reported being affected [57]. Compared to our study, 58% of males and 60% of females reported “Not at all” affected. In the Asia culture, this is a sensitive topic which could be under-reported.

ThyPRO has adequate internal consistency as measured by Cronbach’s alpha of above 0.70 (Table 3). Besides the two items “Pain in front of throat” and “Throat pain felt in ears” in the Goitre symptoms scale, and another two items “Felt in control of life” and “Felt in balance”, in the Emotional scale, the item-scale correlations are above 0.4 (Supplementary I, Table 3) Our well-defined cohort of Graves’ related hyperthyroidism patients may not perceive the importance of these two items in the Goitre scale unlike in subjects with other causes of hyperthyroidism such as subacute thyroiditis which can manifest as tenderness in the thyroid or neck region.

This study supported the hypothesis of construct validity of ThyPRO with good correlative item analysis in Hyperthyroidism symptoms with both clinical symptoms and FT4 concentration (Supplementary I, Table 2). Although a statistical improvement in thyroid volume on 4 months’ follow-up was seen in our cohort, correlative changes in the all the domains of ThyPRO was not seen (Supplementary I, Table 2). This is likely because the evaluation was conducted at just 4 months after initial treatment and the change in thyroid volume was not clinically significant to have cause a change in perceived quality of life.

Our data demonstrated good correlation between the scales (symptoms, psychosocial and Overall QOL Impact) with clinical symptoms and FT4 concentrations. Moreover, the Hyperthyroidism Symptoms scale showed the strongest correlation. This signified convergent validity in these scales.

Similar to a previous study showing at least moderate effect sizes in eight of the scales in ThyPRO (Goiter symptoms, Hyperthyroid symptoms, Tiredness, Anxiety, Depressivity, Emotional susceptibility, Impaired daily life and Overall QOL impact), ThyPRO demonstrated fair responsiveness with moderate effect size in Hyperthyroidism Symptoms scale and small effect sizes in the corresponding scales [27]. This instrument complements physicians’ routine clinical care on clinical signs and symptoms not usually enquired, like subjects’ emotional and low energy status.

The effect sizes of eye symptoms and the cosmetic complaints were low at − 0.1 and 0.1 respectively (Table 4). At baseline, the mean scores for both categories were amongst the lowest. Given the variable presentation and duration of Graves’ thyroid eye manifestations [52, 58], this may explain the minimal improvement in the Eye symptom scale after a short 4 months of anti-thyroid treatment with an effect size of less than the threshold of 0.2. (Table 4) The second reason could be that our patients may not perceive these eye symptoms as important as clinicians [29]. Only 12% of the recruited subjects have eye symptoms on clinical history (Table 1); this mirrored the low prevalence of thyroid eye disease (TED) in our local population [52]. Compared to Caucasians population, our local clinico-epidemiological TED study revealed a lower prevalence of severe TED in Asians; this was attributed to our shallower orbital structure [52, 59]. The third explanation for lower effect size could be that at baseline dry eye symptoms, one of the commonest eye complaints in thyroid eye disease, are very common in our Asian population [52, 59] and so they would not have attributed the symptoms to Graves’ disease. A recently developed and validated Singapore Thyroid Eye Disease QOL (STED-QOL) had similar questions to the Eye component of ThyPRO on its effect on daily activities, getting about, appearance, confidence and work performance affirming our findings on ThyPRO, however STED-QOL has not been tested to look for responsiveness after treatment [60]. The two new questions in STED-QOL specifically addressed eye symptoms not found in ThyPRO were the need to vary head position to have better vision and avoidance of photo-taking because of TED.

Noticeably, the effect size on cosmetic complaints for our local population was minimal. This was unlike a South Korean study evaluating Graves’ ophthalmopathy where most of the subjects were bothered by the change of their appearance (92%) with affected self -esteem (70%). Majority of the Korean subjects (96%) felt that their social wellbeing were affected by their appearance as compared to 70% of them who were affected by their altered vision [61]. Our results are comparable with other Asian communities like Taiwan, and China [62, 63]. Our study population has apparently placed a lower emphasis on their appearances and cosmetics due to Graves’ disease as compared to other aspects of their QOL.

One virtue of this paper is the completeness of data collection with less than 1% of missing data in this study and the characteristics of those who drop-out resembles the remaining subjects. Another strength of our study is that the subject completed the ThyPRO survey on the same day as the physician’s clinical assessment. Our study re-affirms ThyPRO comprehensively in its measurement properties in terms of reliability, validity and responsiveness with its use in a clinic setting. There was no ceiling effect observed with the use of ThyPRO as the developer had used positively worded items thus empowering this tool to discriminate and recognise improvement of study subjects in the QOL on follow-up with serial use of ThyPRO.

We acknowledge the limitations of this study. One drawback of this study is the small sample size given there are 14 scales in this questionnaire. Secondly, the percentage of subjects having the lowest score in the scales: Goitre Symptoms, Eye Symptoms, Cognitive Complaints, Anxiety, Depressivity, Impact on Social, Daily and Sex Life and Overall QoL Impact was above 10 %. We admit that many subjects with the same score may affect the discriminatory ability between subjects and hence affect reliability. Thirdly, only 88% of patients became euthyroid at 4 months. In 4 months’ follow-up, thyroid function changes are too small to fully capture all relevant changes in QOL [21]. Studies have shown persistence of impaired QOL in Graves’ disease patients after treatment for up to 6 months to a year [17, 23].

Our paper looked at the measurement properties of ThyPRO in our local population using a statistical characteristic in a distribution-based approach [64]. To interpret our data in a meaningful manner, we used the last item assessing the negative overall QOL in ThyPRO as the transition question to find out what the Minimally Important Change (M.I.C) is in this global rating that corresponds to the patients’ benefit. We acknowledge that this item did not specifically ask for the improvement in QOL after the commencement of anti-thyroid medication, rather it was based on the QOL over the preceding 4 weeks at 4 months.

When it comes to looking at the QOL, the cross-cultural differences play a key role. As per local practice, anti-thyroid medications are the first line approach towards treatment and are more favoured and used by the endocrinologists. Moving forward, there is a role in the future to study changes with radioactive iodine and surgical treatment. We also need to evaluate the electronic mode of questionnaire administration [65].

## Conclusions

In summary, ThyPRO has satisfactory psychometric properties thus supporting its use for assessing quality of life in Singapore patients with Graves’ hyperthyroidism.

## Supplementary Information

**Additional file 1.**

**Additional file 2.**

## Data Availability

All data generated and analysed during this study are included in this published article (and its supplementary information files).

## Abbreviations

CI: Confidence Interval
DSRB: Domain Specific Review Board
ES: Effect Size
FT4: Free thyroxine
GRASS: Graves’ disease selenium supplementation trial
HCQ: Hyperthyroid complaints questionnaire
IQR: Interquartile range
IRB: Institutional review board
IU: International Unit
MIC: Minimally Important Change
mIU: Milli international Unit
PRO: Patient Reported Outcome
QOL: Quality of life
RCT: Randomized clinical trial
SD: Standard Deviation
TFT: Thyroid function test
ThyPRO: Thyroid- related patient reported outcome
TRAb: TSH receptor antibody
TSH: Thyrotropin/ Thyroid stimulating hormone
TSI: Thyroid Stimulating Immunoglobulin
