# Supplementary material for: Psychometric properties of the thyroid-specific quality of life questionnaire ThyPRO in Singaporean patients with Graves’ disease
Source: J Patient Rep Outcomes. 2021 Jul 8;5:54. doi: 10.1186/s41687-021-00309-x (PMC8266927; doi:10.1186/s41687-021-00309-x)
Supplement: Supplementary file 1 — Additional file 1. [file 41687_2021_309_MOESM1_ESM.docx]

**Supplementary I**

**Table 1: Electronic template for Hyperthyroid patients**

| **Symptoms (Clinician’s review in template)** | |
| --- | --- |
| General status | Irritable or anxious lately |
|  | Heat intolerance |
| Cardiorespiratory | Palpitations |
|  | Chest pain on exertion |
|  | Symptoms of Congestive Cardiac Failure |
| Gastrointestinal | Lost weight unintentionally |
|  | Increased appetite |
|  | Increased bowel motion frequency |
| Neurological | Tremors |
|  | Lower limb weakness after heavy meals |
| Eyes | Grittiness/ dry |
|  | Puffiness/red |
|  | Double vision |
|  | Blurring of vision |
| Constitutional | Fever |
|  | Upper respiratory infection/ intercurrent illnesses |

**Table 2: Construct validity Correlation Analysis**

| **Scale** | **Symptoms** | | | | | | **Biochemical and US** | | | |
| --- | --- | --- | --- | --- | --- | --- | --- | --- | --- | --- |
|  | **General** | **Cardiorespiratory** | **Gastrointestinal** | **Neurological** | **Eye** | **Constitutional** | **Free thyroxine, FT4** | **Thyrotropin, TSH** | **Thyroid Receptor Antibody, TRAB** | **Thyroid Volume** |
| **ThyPRO** | | | | | | | | | | |
| *Symptoms* | | | | | | | | | | |
| Goitre | -0.046 | 0.210^α^ | 0.110 | 0.095 | 0.034 | 0.095 | 0.417^¶^ | -0.178 | -0.131 | 0.123 |
| Hyperthyroid | 0.255^α^ | 0.351^α^ | 0.314^α^ | 0.347^α^ | 0.245^α^ | 0.076 | 0.636* | -0.157 | 0.170^α^ | -0.065 |
| Eye | 0.080 | 0.118 | 0.308^α^ | 0.298^α^ | 0.279^α^ | -0.126 | 0.299^α^ | -0.162 | 0.153 | -0.083 |
| *Social and functional well-being* | | | | | | | | | | |
| Tiredness | -0.051 | 0.226^α^ | 0.221^α^ | 0.096 | 0.178 | 0.228^α^ | 0.314^α^ | -0.235 | 0.042 | -0.027 |
| Cognitive | 0.034 | 0.368^¶^ | 0.246^α^ | 0.169 | 0.156 | -0.021 | 0.358^¶^ | -0.106 | 0.108 | 0.105 |
| Anxiety | 0.074 | 0.348^α^ | 0.173 | 0.159 | 0.144 | 0.119 | 0.449^¶^ | -0.084 | 0.122 | 0.019 |
| Depression | -0.033 | 0.112 | 0.086 | -0.016 | 0.014 | 0.122 | 0.325^α^ | -0.065 | -0.010 | 0.044 |
| Emotional | 0.030 | 0.160 | 0.064 | 0.016 | 0.209 ^α^ | -0.167 | 0.307^α^ | -0.099 | 0.034 | 0.119 |
| Impaired social life | -0.005 | 0.212 ^α^ | 0.140 | 0.132 | 0.247^α^ | -0.040 | 0.373^¶^ | -0.083 | -0.045 | 0.109 |
| Impaired daily life | -0.069 | 0.278^α^ | 0.111 | 0.125 | -0.113 | 0.147 | 0.361^¶^ | -0.122 | -0.079 | -0.043 |
| Impaired sex life | -0.280 | 0.061 | 0.041 | -0.072 | -0.231 | 0.123 | 0.205^α^ | -0.041 | -0.001 | -0.041 |
| Cosmetic | -0.057 | 0.245^α^ | 0.151 | -0.063 | 0.147 | -0.021 | 0.300^α^ | -0.060 | 0.174^α^ | 0.063 |
| Negative QoL | -0.077 | 0.236^α^ | 0.005 | 0.075 | -0.038 | 0.034 | 0.325^α^ | -0.091 | -0.014 | -0.086 |
| * P value < 0.05  A correlative coefficient value of 0.5* or higher, 0.35-0.5^¶^, and 0.2 – 0.35^α^ would be considered strong, moderate, and weak correlation respectively. | | | | | | | | | | |

**Table 3: Item-Scale Correlation ThyPRO**

|  | Goitre | Hyper  thyroid | Hypo  thyroid | Eye | Tired | Cognitive | Anxiety | Depress | Emotional | Imp  Social | Imp  Daily | Imp  Sex | Cosmetic | Neg  QOL |
| --- | --- | --- | --- | --- | --- | --- | --- | --- | --- | --- | --- | --- | --- | --- |
| **Goitre** |  |  |  |  |  |  |  |  |  |  |  |  |  |  |
| Fullness in the neck | 0.60* | 0.19 | 0.35 | 0.00 | 0.40 | 0.36 | 0.17 | 0.24 | 0.21 | 0.17 | 0.35 | 0.25 | 0.36 | 0.31 |
| Visible swelling in front of neck | 0.56* | 0.17 | 0.33 | 0.12 | 0.30 | 0.45 | 0.16 | 0.21 | 0.30 | 0.23 | 0.31 | 0.10 | 0.32 | 0.30 |
| Pressure in throat | 0.68* | 0.29 | 0.16 | 0.20 | 0.48 | 0.27 | 0.30 | 0.21 | 0.25 | 0.16 | 0.38 | 0.15 | 0.19 | 0.28 |
| Pain in front of throat | 0.29 | 0.01 | -0.11 | 0.16 | 0.29 | 0.01 | -0.02 | 0.18 | 0.14 | -0.01 | 0.09 | 0.10 | 0.07 | 0.15 |
| Throat pain felt in ears | 0.13 | 0.29 | 0.29 | 0.31 | 0.28 | 0.26 | 0.37 | 0.27 | 0.36 | 0.29 | 0.30 | 0.26 | 0.37 | 0.24 |
| Lump in throat | 0.59* | 0.23 | 0.15 | 0.02 | 0.28 | 0.10 | 0.19 | 0.14 | 0.29 | 0.21 | 0.20 | 0.10 | 0.21 | 0.15 |
| Clearing throat often | 0.71* | 0.46 | 0.39 | 0.30 | 0.56* | 0.28 | 0.40 | 0.35 | 0.40 | 0.36 | 0.43 | 0.05 | 0.36 | 0.48 |
| Discomfort swallowing | 0.51 | 0.12 | -0.04 | 0.02 | 0.26 | 0.02 | 0.03 | 0.04 | 0.21 | 0.10 | 0.23 | 0.14 | 0.09 | 0.19 |
| Difficulty swallowing | 0.49 | 0.24 | 0.04 | 0.23 | 0.31 | 0.11 | 0.05 | 0.00 | 0.23 | 0.06 | 0.34 | 0.00 | 0.21 | 0.39 |
| Sense of suffocating | 0.57* | 0.28 | 0.34 | 0.19 | 0.39 | 0.39 | 0.24 | 0.24 | 0.28 | 0.24 | 0.48 | 0.27 | 0.19 | 0.48 |
| Hoarseness | 0.51 | 0.22 | 0.17 | 0.14 | 0.35 | 0.30 | 0.20 | 0.14 | 0.27 | 0.17 | 0.43 | 0.19 | 0.26 | 0.40 |
| **Hyperthyroid Symptom** |  |  |  |  |  |  |  |  |  |  |  |  |  |  |
| Trembling hands | 0.34 | 0.72* | 0.55* | 0.44 | 0.36 | 0.32 | 0.39 | 0.31 | 0.32 | 0.39 | 0.17 | 0.21 | 0.41 | 0.34 |
| Increased sweating | 0.28 | 0.82* | 0.43 | 0.50 | 0.34 | 0.37 | 0.37 | 0.36 | 0.34 | 0.44 | 0.33 | 0.18 | 0.44 | 0.50* |
| Palpitations ( Rapid heartbeat) | 0.41 | 0.81* | 0.38 | 0.48 | 0.45* | 0.34 | 0.59* | 0.48 | 0.33 | 0.35 | 0.47 | 0.29 | 0.44 | 0.54* |
| Shortness of breath | 0.51* | 0.78* | 0.50* | 0.43 | 0.62* | 0.50* | 0.59* | 0.52* | 0.51* | 0.43 | 0.53* | 0.19 | 0.35 | 0.58* |
| Sensitive to heat | 0.48 | 0.68* | 0.53* | 0.50 | 0.37 | 0.46 | 0.41 | 0.30 | 0.34 | 0.41 | 0.43 | 0.22 | 0.41 | 0.49 |
| Increased appetite | 0.26 | 0.68* | 0.50* | 0.32 | 0.22 | 0.22 | 0.38 | 0.27 | 0.28 | 0.32 | 0.14 | 0.12 | 0.44 | 0.19 |
| Had loose stools | 0.13 | 0.55* | 0.34 | 0.27 | 0.39 | 0.15 | 0.39 | 0.26 | 0.09 | 0.29 | 0.29 | 0.15 | 0.21 | 0.13 |
| Had upset stomach | 0.29 | 0.64* | 0.43 | 0.40 | 0.47 | 0.20 | 0.32 | 0.48 | 0.24 | 0.26 | 0.37 | 0.10 | 0.45 | 0.44 |
| **Hypothyroid Symptoms** |  |  |  |  |  |  |  |  |  |  |  |  |  |  |
| Sensitive to cold | 0.62* | 0.42 | 0.59* | 0.51* | 0.52* | 0.57* | 0.31 | 0.37 | 0.55* | 0.34 | 0.43 | 0.13 | 0.49 | 0.47 |
| Swollen hands or feet | 0.38 | 0.34 | 0.71* | 0.47 | 0.59* | 0.64* | 0.45 | 0.54* | 0.40 | 0.42 | 0.65* | 0.45 | 0.44 | 0.52* |
| Dry skin | 0.19 | 0.27 | 0.65* | 0.38 | 0.26 | 0.38 | 0.41 | 0.28 | 0.48 | 0.48 | 0.34 | 0.42 | 0.33 | 0.21 |
| Itchy skin | 0.33 | 0.48 | 0.80* | 0.43 | 0.48 | 0.56* | 0.51* | 0.58* | 0.41 | 0.54* | 0.46 | 0.37 | 0.42 | 0.44 |
|  | **Goitre** | **Hyper**  **thyroid** | **Hypo**  **thyroid** | **Eye** | **Tired** | **Cognitive** | **Anxiety** | **Depress** | **Emotional** | **Imp**  **Social** | **Imp**  **Daily** | **Imp**  **Sex** | **Cosmetic** | **Neg**  **QOL** |
| **Eye** |  |  |  |  |  |  |  |  |  |  |  |  |  |  |
| Moist or watery eyes | 0.33 | 0.41 | 0.38 | 0.75* | 0.58* | 0.41 | 0.39 | 0.43 | 0.50 | 0.38 | 0.45 | 0.21 | 0.32 | 0.50* |
| Bags under the eyes | 0.35 | 0.54* | 0.51* | 0.79* | 0.43 | 0.43 | 0.26 | 0.29 | 0.37 | 0.33 | 0.31 | 0.29 | 0.50 | 0.44 |
| Dryness or “grittiness” in eyes | 0.14 | 0.51* | 0.59* | 0.87* | 0.45 | 0.51* | 0.36 | 0.44 | 0.38 | 0.36 | 0.39 | 0.19 | 0.35 | 0.39 |
| Impaired vision | 0.14 | 0.12 | 0.25 | 0.51 | 0.46 | 0.46 | 0.32 | 0.33 | 0.28 | 0.21 | 0.39 | 0.22 | 0.13 | 0.29 |
| Pressure in (or behind) eyes | 0.28 | 0.35 | 0.47 | 0.74* | 0.43 | 0.43 | 0.36 | 0.35 | 0.36 | 0.34 | 0.39 | 0.26 | 0.31 | 0.35 |
| Double vision | 0.09 | 0.24 | 0.30 | 0.60* | 0.30 | 0.39 | 0.17 | 0.27 | 0.16 | 0.13 | 0.29 | 0.17 | 0.19 | 0.33 |
| Eye pain | 0.04 | 0.22 | 0.24 | 0.52* | 0.29 | 0.31 | 0.30 | 0.36 | 0.34 | 0.20 | 0.24 | 0.11 | 0.22 | 0.32 |
| Very sensitive to light | 0.42 | 0.57* | 0.63* | 0.74* | 0.58* | 0.71* | 0.48 | 0.63* | 0.56* | 0.54* | 0.54* | 0.36 | 0.47 | 0.51 |
| **Tired** |  |  |  |  |  |  |  |  |  |  |  |  |  |  |
| Tiredness | 0.59* | 0.53* | 0.60* | 0.48 | 0.86* | 0.57* | 0.44 | 0.39 | 0.44 | 0.45 | 0.65* | 0.43 | 0.56* | 0.49 |
| Exhaustion | 0.55* | 0.61* | 0.61* | 0.57* | 0.85* | 0.60* | 0.55* | 0.47 | 0.52* | 0.55* | 0.71* | 0.43 | 0.56* | 0.59* |
| Difficulty getting motivated | 0.56* | 0.47 | 0.52* | 0.48 | 0.77* | 0.62* | 0.65* | 0.51* | 0.51* | 0.66* | 0.74* | 0.40 | 0.40 | 0.60* |
| Worn out | 0.56* | 0.55* | 0.66* | 0.52* | 0.81* | 0.61* | 0.51* | 0.42 | 0.58* | 0.57* | 0.68* | 0.41 | 0.60* | 0.61* |
| Full of life | 0.37 | 0.27 | 0.43 | 0.47 | 0.72* | 0.31 | 0.28 | 0.50 | 0.32 | 0.22 | 0.35 | 0.26 | 0.26 | 0.36 |
| Energetic | 0.19 | 0.14 | 0.32 | 0.35 | 0.67* | 0.24 | 0.12 | 0.47 | 0.24 | 0.17 | 0.27 | 0.27 | 0.34 | 0.30 |
| Able to cope with life | 0.18 | 0.07 | -0.01 | 0.14 | 0.39 | 0.11 | 0.00 | 0.37 | 0.14 | -0.08 | 0.14 | 0.15 | -0.01 | 0.19 |
| **Cognition** |  |  |  |  |  |  |  |  |  |  |  |  |  |  |
| Difficulty remembering | 0.28 | 0.32 | 0.55* | 0.41 | 0.43 | 0.89* | 0.42 | 0.34 | 0.50* | 0.52* | 0.55* | 0.45 | 0.46 | 0.44 |
| Slow or unclear thinking | 0.47 | 0.39 | 0.67* | 0.52* | 0.63* | 0.90* | 0.49 | 0.55* | 0.63* | 0.57* | 0.69* | 0.50* | 0.44 | 0.56* |
| Difficulty finding the right words | 0.46 | 0.36 | 0.56* | 0.54* | 0.61* | 0.82* | 0.43 | 0.49 | 0.49 | 0.52* | 0.64* | 0.41 | 0.32 | 0.60* |
| Confusion | 0.45 | 0.37 | 0.55* | 0.56* | 0.60* | 0.82* | 0.50* | 0.55* | 0.58* | 0.51* | 0.62* | 0.29 | 0.34 | 0.58* |
| Difficulty learning new | 0.31 | 0.38 | 0.48 | 0.41 | 0.52* | 0.78* | 0.41 | 0.36 | 0.37 | 0.36 | 0.53* | 0.36 | 0.28 | 0.53* |
| Difficulty concentrating | 0.52* | 0.49 | 0.68* | 0.60* | 0.63* | 0.88* | 0.46 | 0.57* | 0.60* | 0.49 | 0.66* | 0.44 | 0.51* | 0.67* |
|  |  |  |  |  |  |  |  |  |  |  |  |  |  |  |
|  | **Goitre** | **Hyper**  **thyroid** | **Hypo**  **thyroid** | **Eye** | **Tired** | **Cognitive** | **Anxiety** | **Depress** | **Emotional** | **Imp**  **Social** | **Imp**  **Daily** | **Imp**  **Sex** | **Cosmetic** | **Neg**  **QOL** |
| **Anxiety** |  |  |  |  |  |  |  |  |  |  |  |  |  |  |
| Nervous | 0.43 | 0.53* | 0.52* | 0.38 | 0.32 | 0.55* | 0.89* | 0.48 | 0.54* | 0.62* | 0.49 | 0.24 | 0.32 | 0.49 |
| Anxious | 0.51* | 0.61* | 0.63* | 0.51* | 0.49 | 0.60* | 0.88* | 0.61* | 0.68* | 0.58* | 0.56* | 0.30 | 0.44 | 0.52* |
| Tense | 0.41 | 0.51* | 0.64* | 0.47 | 0.49 | 0.65* | 0.85* | 0.49 | 0.56* | 0.62* | 0.54* | 0.38 | 0.29 | 0.47 |
| Concerned about being seriously ill | 0.26 | 0.34 | 0.32 | 0.22 | 0.25 | 0.16 | 0.67* | 0.32 | 0.31 | 0.34 | 0.42 | 0.41 | 0.13 | 0.32 |
| Uneasy | 0.45 | 0.56* | 0.56* | 0.46 | 0.67* | 0.37 | 0.71* | 0.76* | 0.56* | 0.50* | 0.52* | 0.42 | 0.41 | 0.54* |
| Restless | 0.45 | 0.55* | 0.49 | 0.32 | 0.57* | 0.50 | 0.76* | 0.52* | 0.48 | 0.58* | 0.54* | 0.22 | 0.34 | 0.57* |
| **Depressivity** |  |  |  |  |  |  |  |  |  |  |  |  |  |  |
| Sad | 0.39 | 0.54* | 0.65* | 0.57* | 0.47 | 0.60* | 0.59* | 0.86* | 0.59* | 0.53* | 0.56* | 0.46 | 0.60* | 0.67* |
| Depressed | 0.28 | 0.46 | 0.42 | 0.36 | 0.41 | 0.34 | 0.67* | 0.77* | 0.48 | 0.49 | 0.54* | 0.36 | 0.34 | 0.61* |
| Discouraged | 0.39 | 0.53* | 0.51* | 0.48 | 0.53* | 0.49 | 0.62* | 0.79* | 0.50* | 0.50* | 0.73* | 0.35 | 0.50* | 0.75* |
| Cried easily | 0.39 | 0.49 | 0.63* | 0.45 | 0.46 | 0.59* | 0.62* | 0.77* | 0.61* | 0.60* | 0.64* | 0.41 | 0.59* | 0.61* |
| Unhappy | 0.43 | 0.46 | 0.56* | 0.41 | 0.43 | 0.50* | 0.66* | 0.85* | 0.67* | 0.59* | 0.56* | 0.42 | 0.47 | 0.67* |
| Happy | 0.23 | 0.18 | 0.27 | 0.12 | 0.53* | 0.30 | 0.24 | 0.66* | 0.27 | 0.20 | 0.30 | 0.28 | 0.16 | 0.31 |
| Self-confident | 0.28 | 0.26 | 0.18 | 0.15 | 0.42 | 0.15 | 0.19 | 0.64* | 0.14 | 0.18 | 0.26 | 0.18 | 0.14 | 0.28 |
| **Emotional** |  |  |  |  |  |  |  |  |  |  |  |  |  |  |
| Difficulty coping | 0.46 | 0.34 | 0.42 | 0.31 | 0.50* | 0.55* | 0.56* | 0.48 | 0.61* | 0.60* | 0.56* | 0.36 | 0.44 | 0.55* |
| Feeling “not like oneself” | 0.60* | 0.48 | 0.47 | 0.38 | 0.55* | 0.42 | 0.51* | 0.54* | 0.71* | 0.52* | 0.43 | 0.29 | 0.52* | 0.57* |
| Easily feeling stressed | 0.54* | 0.53* | 0.65* | 0.52* | 0.58* | 0.65* | 0.62* | 0.50* | 0.83* | 0.56* | 0.60* | 0.36 | 0.57* | 0.66* |
| Mood swings | 0.52* | 0.50* | 0.65* | 0.57* | 0.50 | 0.72* | 0.54* | 0.49 | 0.80* | 0.54* | 0.48 | 0.33 | 0.64* | 0.60* |
| Irritable | 0.56* | 0.49 | 0.65* | 0.47 | 0.48 | 0.63* | 0.67* | 0.50* | 0.81* | 0.58* | 0.38 | 0.24 | 0.49 | 0.49 |
| Frustrated | 0.46 | 0.43 | 0.61* | 0.33 | 0.40 | 0.55* | 0.65* | 0.44 | 0.85* | 0.55* | 0.41 | 0.35 | 0.41 | 0.49 |
| Angry | 0.45 | 0.49 | 0.57* | 0.40 | 0.41 | 0.57* | 0.59* | 0.48 | 0.83* | 0.60* | 0.44 | 0.32 | 0.47 | 0.49 |
| In control of life | -0.14 | -0.30 | -0.23 | -0.11 | 0.09 | -0.11 | -0.22 | 0.12 | 0.08 | -0.21 | -0.07 | 0.16 | -0.16 | -0.10 |
| In balance | -0.22 | -0.40 | -0.29 | -0.15 | 0.05 | -0.19 | -0.36 | -0.01 | -0.02 | -0.36 | -0.17 | 0.04 | -0.15 | -0.18 |
|  |  |  |  |  |  |  |  |  |  |  |  |  |  |  |
|  | **Goitre** | **Hyper**  **thyroid** | **Hypo**  **thyroid** | **Eye** | **Tired** | **Cognitive** | **Anxiety** | **Depress** | **Emotional** | **Imp**  **Social** | **Imp**  **Daily** | **Imp**  **Sex** | **Cosmetic** | **Neg**  **QOL** |
| **Impact on Social life** |  |  |  |  |  |  |  |  |  |  |  |  |  |  |
| Difficult being with other people | 0.46 | 0.55* | 0.62* | 0.43 | 0.64* | 0.67* | 0.55* | 0.68* | 0.52* | 0.74* | 0.62* | 0.44 | 0.47 | 0.58* |
| A burden to other people | 0.38 | 0.27 | 0.40 | 0.18 | 0.42 | 0.27 | 0.43 | 0.39 | 0.33 | 0.73* | 0.56* | 0.48 | 0.32 | 0.32 |
| Conflicts with other people | 0.33 | 0.42 | 0.42 | 0.23 | 0.24 | 0.20 | 0.37 | 0.44 | 0.40 | 0.66* | 0.25 | 0.14 | 0.24 | 0.33 |
| People lack understanding | 0.38 | 0.34 | 0.38 | 0.35 | 0.30 | 0.41 | 0.46 | 0.26 | 0.44 | 0.77* | 0.34 | 0.37 | 0.36 | 0.35 |
| **Impact on Daily Living** |  |  |  |  |  |  |  |  |  |  |  |  |  |  |
| Difficulty managing daily life | 0.47 | 0.46 | 0.41 | 0.36 | 0.53* | 0.52* | 0.43 | 0.45 | 0.37 | 0.55* | 0.79* | 0.57* | 0.40 | 0.68* |
| Limit leisure activities | 0.61* | 0.45 | 0.54* | 0.37 | 0.65* | 0.61* | 0.52* | 0.48 | 0.45 | 0.57* | 0.88* | 0.60* | 0.41 | 0.65* |
| Difficulty participating in life | 0.56* | 0.37 | 0.53* | 0.38 | 0.63* | 0.60* | 0.46 | 0.43 | 0.47 | 0.50 | 0.87* | 0.58* | 0.46 | 0.69* |
| Difficulty getting around | 0.49 | 0.32 | 0.41 | 0.39 | 0.70* | 0.60* | 0.48 | 0.52* | 0.42 | 0.47 | 0.89* | 0.50* | 0.39 | 0.75* |
| Everything takes longer | 0.44 | 0.32 | 0.33 | 0.35 | 0.62* | 0.49 | 0.44 | 0.46 | 0.34 | 0.41 | 0.86* | 0.41 | 0.34 | 0.75* |
| Difficulty managing job | 0.32 | 0.39 | 0.37 | 0.41 | 0.52* | 0.55* | 0.47 | 0.52* | 0.31 | 0.48 | 0.74* | 0.48 | 0.46 | 0.63* |
| **Impact on Sex life** |  |  |  |  |  |  |  |  |  |  |  |  |  |  |
| Negative influence on sex life | 0.25 | 0.20 | 0.33 | 0.25 | 0.39 | 0.37 | 0.34 | 0.35 | 0.38 | 0.44 | 0.47 | 0.94* | 0.36 | 0.38 |
| Decreased sexual desire | 0.29 | 0.26 | 0.43 | 0.24 | 0.39 | 0.45 | 0.37 | 0.39 | 0.35 | 0.44 | 0.56* | 1.00* | 0.37 | 0.44 |
| **Cosmetic** |  |  |  |  |  |  |  |  |  |  |  |  |  |  |
| Disease affect appearance | 0.36 | 0.57* | 0.51* | 0.47 | 0.52* | 0.44 | 0.27 | 0.49 | 0.37 | 0.35 | 0.44 | 0.35 | 0.89* | 0.57* |
| Unsatisfied with appearance | 0.31 | 0.58* | 0.45 | 0.46 | 0.41 | 0.43 | 0.34 | 0.42 | 0.43 | 0.29 | 0.39 | 0.25 | 0.84* | 0.65* |
| Camouflage or mask visible signs | 0.17 | 0.26 | 0.41 | 0.42 | 0.35 | 0.34 | 0.16 | 0.32 | 0.30 | 0.40 | 0.35 | 0.47 | 0.58* | 0.35 |
| Bothered by other people looking | 0.19 | 0.38 | 0.44 | 0.51* | 0.32 | 0.34 | 0.28 | 0.37 | 0.38 | 0.36 | 0.36 | 0.23 | 0.72* | 0.36 |
| Influence on clothes worn | 0.22 | 0.23 | 0.50* | 0.37 | 0.36 | 0.46 | 0.37 | 0.48 | 0.51* | 0.42 | 0.40 | 0.24 | 0.55* | 0.34 |
| Felt too fat | 0.03 | 0.05 | 0.11 | 0.10 | 0.03 | 0.10 | -0.04 | 0.04 | 0.28 | 0.16 | 0.08 | 0.08 | 0.52* | 0.14 |
| **Negative QoL** | 0.56* | 0.55* | 0.49* | 0.50* | 0.62* | 0.63* | 0.52* | 0.66* | 0.62* | 0.51* | 0.81* | 0.44 | 0.61* | 1.00* |
