# Supplementary material for: Psychometric properties of the thyroid-specific quality of life questionnaire ThyPRO in Singaporean patients with Graves’ disease
Source: J Patient Rep Outcomes. 2021 Jul 8;5:54. doi: 10.1186/s41687-021-00309-x (PMC8266927; doi:10.1186/s41687-021-00309-x)
Supplement: Supplementary file 2 — Additional file 2. [file 41687_2021_309_MOESM2_ESM.docx]

| Supplementary II |
| --- |
| Figure 1: Histograms showing the percentage of patients achieving baseline ThyPRO subscales score (0–100). |
| Baseline |
|   Goitre Scale Score _Baseline, range 0-45, F/C 20/0 |
|   Hyperthyroidism symptoms scale score _Baseline, range 0-78, F/C 4/0 |
|   Hypothyroidism symptoms scale score_ baseline, range 0-100, F/C 17/2 |
|   Eye symptoms scale score _ baseline, range 0-75, F/C 33/0 |
|   Tired symptoms scale score _baseline, range 0-100, F/C 2/4 |
|   Cognitive symptoms scale _baseline, range 0-100, F/C 31/4 |
|   Anxiety symptoms scale _baseline, range 0-100, F/C 17/2 |
|   Depressivity symptoms scale _baseline, range 0-100, F/C 10/2 |
|   Emotional symptom scale_ baseline, range 3-100, F/C 0/2 |
|   Impact on Social life Scale Score _ baseline, range 0-100, F/C 25/2 |
|   Impact on Daily Life Scale Score _ baseline, range 0-100, F/C 40/2 |
|   Impact on Sex Life Scale Score _ baseline, range 0-100, 60/6 |
|   Cosmetic Scale Score _ baseline, range 0-96, F/C 25/0 |
|   Negative QOL Scale Score _ baseline, range 0-100, F/C 31/13 |
| Legend: Scores- numerical values. F – Floor effect with minimum possible score of 0; C – Ceiling effect with maximum possible score of 100. |

Figure 2a: Boxplot of the items in Goitre scale against the response categories

Figure 2b: Boxplot of the items in Hypothyroid symptom scale against the response categories

Figure 2c: Boxplot of the items in Eye symptoms scale against the response categories

Figure 2d: Boxplot of the items in Cognitive symptoms scale against the response categories

Figure 2e: Boxplot of the items in Anxiety scale against the response categories

Figure 2f: Boxplot of the items in Impact on Social Life scale against the response categories

Figure 2g: Boxplot of the items in Impact on Daily life scale against the response categories

Figure 2h: Boxplot of the items in Impact on Sex Life scale against the response categories

Figure 2i: Boxplot of the items in Cosmetic scale against the response categories

Figure 2j: Boxplot of the items in Negative QOL scale against the response categories

Figure 3a: Mean change of Negative QOL score based on baseline Negative QOL categories


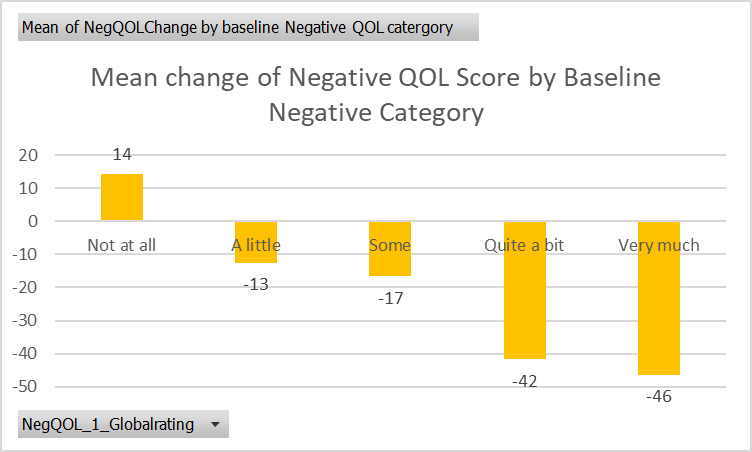


Figure 3b: Mean change of Negative QOL score based on QOL categories at 4 months
